# Effects of water and fertilizer coupling on growth and development of Chinese dwarf cherry (*Cerasus humilis*) and evaluation of water and fertilizer use efficiency

**DOI:** 10.3389/fpls.2026.1841934

**Published:** 2026-05-18

**Authors:** Shuang Liu, Zhongyang Li, Shanshan Chen, Yue Yu, Siyu Wang, Lei Zhang, Jinying Li, Shuang Li, Xuedong Tang, Jianlei Qiao

**Affiliations:** 1 College of Horticulture, Jilin Agricultural University, Changchun, China; 2 College of Biological and Agricultural Engineering, Jilin University, Changchun, China; 3 Management Office of Teaching and Scientific Research Base, Jilin Agricultural University, Changchun, China

**Keywords:** Chinese dwarf cherry (*Cerasus humilis*), growth, photosynthetic characteristics, quality, water and fertilizer coupling, water and fertilizer use efficiency, yield

## Abstract

**Introduction:**

In recent years, the demand for high-nutrition fruits has increased. As a unique fruit tree in China, *Cerasus humilis* has both ecological and nutritional value. Water and fertilizer nutrition is closely related to plant growth and development.

**Methods:**

In this study, ‘ Nongda No.6 ‘ *Cerasus humilis* was used as a test material to carry out a field drip fertigation experiment to study the comprehensive effects of water and fertilizer coupling regulation on the growth, photosynthesis, yield, quality and water and fertilizer use efficiency of *Cerasus humilis*, and a water and fertilizer coupling control scheme suitable for the region was proposed. The irrigation amount was set at three levels: W1 (60%FC), W2 (70%FC) and W3 (80%FC). The amount of compound fertilizer was set at three levels, F1(66kg/667m^2^), F2(99kg/667m^2^), F3(132kg/667m^2^), a total of 9 treatments.

**Results and discussion:**

The results showed that under the combination of irrigation amount of W2(70%FC) and fertilization amount of F2(99kg/667m^2^), the growth effect of *Cerasus humilis* was better, and the net photosynthetic rate and transpiration rate of leaves were at a better level during the whole growth period. In addition, the fruit yield of *Cerasus humilis* under W2F2 treatment was the highest, reaching the highest value of 1109kg/667m^2^, and the fruit quality was ideal, the contents of soluble sugar and soluble protein were 7.14% and 4.25 mg/g, the vitamin C content and solid acid ratio (soluble solids/titratable acid) reached the maximum under W2F3 treatment. The IWUE and PFP of *Cerasus humilis* under W2F2 treatment were also at a better level, which were 6.14kg/mm·667m² and 11.21, respectively. The results of comprehensive analysis showed that although W2F3 had advantages in vitamin C content and solid acid ratio, W2F2 provided the best comprehensive balance between yield, soluble sugar, soluble protein and water and fertilizer use efficiency. In summary, W2F2 with the best comprehensive performance is recommended as a water and fertilizer management scheme for efficient production of *Cerasus humilis*.

## 1 Introduction

*Cerasus humilis* (Bge.) Sok., a small, perennial, deciduous shrub belonging to the Rosaceae family of fruit trees, and it is a unique fruit tree resource in China. It is mainly distributed in northern China (Pan et al., 2025), including Shanxi, Inner Mongolia, Liaoning, and Hebei, and has existed in nature for a long time. Because of its high calcium content, it is also known as ‘calcium fruit’ (Wang et al., 2019). At the same time, *Cerasus humilis* fruit contains a variety of minerals, has a high nutritional value (Song et al., 2011), and is rich in sugar, vitamins, organic acids, and other nutrients (Liu et al., 2023). Its roots develop well, giving it strong ecological adaptability to cold, drought, abiotic stress, and saline-alkali soils. It is an excellent species for windbreaks and sand fixation and has broad prospects for research and utilization (Li et al., 2014).

The growth and development, yield and quality of *Cerasus humilis* depend on many environment-related variables, among which water and fertilizer are two important factors. In the traditional planting process, excessive irrigation and fertilization are common, resulting in a waste of water and fertilizer resources and a decrease in crop yield (Wang et al., 2022), in this context, water-saving irrigation technology has been continuously developed. Relevant studies have systematically reviewed the effects of different irrigation methods on crop water and fertilizer use efficiency and emphasized the role of water regulation in sustainable agricultural production (Franco-Navarro et al., 2025), therefore, reasonable regulation of water and fertilizer is an important way to improve crop yield and quality (Wang et al., 2018; Liu et al., 2019). At present, water and fertilizer coupling technology has been widely used in the cultivation of various types of fruit trees and woody plants. Relevant research has clarified its regulatory effect on plant growth, development, physiological mechanism and water and fertilizer utilization rate (Ma, 2023; Dai et al., 2019). It is very important to improve fruit quality, crop photosynthetic capacity and crop water and fertilizer use efficiency. Li et al. showed that (Li et al., 2024), appropriate water and fertilizer coupling treatment has a positive effect on improving the growth and physiological characteristics of young apple trees, it can improve the plant growth, basal stem growth, dry matter, photosynthetic rate, stomatal conductance and water use efficiency of young apple trees and realize the coordinated development of high yield and water saving and fertilizer saving. Liu et al. ‘ s water and fertilizer coupling experiment on jujube showed that (Liu et al., 2017), water and fertilizer interaction significantly increased fruit yield and water and nitrogen use efficiency. Therefore, the study of water and fertilizer coupling has important theoretical and practical significance for the design of efficient and high-yield crop irrigation and fertilization systems, these systems can save limited resources and are cost-effective and sustainable; these studies provide a solid theoretical basis and practical reference for the application of water and fertilizer coupling technology, but the biological characteristics, growth needs and cultivation environment of different tree species are different, and the suitable parameters and regulation mechanism of water and fertilizer coupling also show species specificity.

With the intensification of *Cerasus humilis* cultivation, the irrigation and fertilization technology of *Cerasus humilis* has been developed, but most studies focus on the effects of single water or nutrient factors on the growth, yield and quality of *Cerasus humilis* (Yin et al., 2016), there are also a small number of studies involving the effects of water and fertilizer coupling on the growth, physiology and yield of *Cerasus humilis*, and some suitable water and fertilizer combination schemes are preliminarily screened out (Zhang et al., 2018). But there are still problems, furrow irrigation and flood irrigation are the main irrigation methods, and drip irrigation is tried in some production areas, however, there are some problems, such as unreasonable irrigation quota setting, mismatch between timing and water demand law of *Cerasus humilis*, and non-standard application of drip irrigation system (Bian et al., 2017). Fertilization is mainly based on furrow application. At present, there is a lack of research on the special fertilization ratio of *Cerasus humilis*, unreasonable nutrient input, low precision fertilization level, and insufficient scientific and targeted fertilization scheme (Wang et al., 2025; Ding et al., 2022). Compared with other woody fruit trees, the research on water and fertilizer coupling of *Cerasus humilis* started late, and has not yet formed a complete theoretical system and technical specifications, most cultivations lack systematic exploration of how water and fertilizer coupling synergistically regulates key indicators such as photosynthetic characteristics, growth and development, yield and quality, and water and fertilizer utilization rate of *Cerasus humilis*, and the regulation of water and fertilizer coupling on these indicators is not fully elucidated. The existing research has not fully combined the biological characteristics of *Cerasus humilis* with the environmental characteristics of different cultivation areas, and the research on the optimization of water and fertilizer coupling parameters is not deep enough, it is impossible to provide accurate water and fertilizer control schemes for *Cerasus humilis* cultivation in different production areas, and cannot meet the current needs of intensive, high-quality and efficient cultivation of *Cerasus humilis* (Zhang et al., 2018).

In summary, although there are many studies on water and fertilizer coupling, most of them focus on other crops, and the research on water and fertilizer management of *Cerasus humilis* focuses on the effect of single factor, failing to fully consider the synergistic interaction and coupling effect between water and fertilizer. Therefore, in this study, *Cerasus humilis* was used as the experimental material, and different water and fertilizer combination treatments were set up to systematically explore the regulation effect of water and fertilizer coupling (drip irrigation + NPK compound fertilizer) on the growth, photosynthesis, yield, quality and resource use efficiency of *Cerasus humilis*. The purpose of this study is to screen out the high-efficiency water and fertilizer combination scheme suitable for the large-scale production of *Cerasus humilis*, and to provide reference for the high-efficiency water and fertilizer management scheme needed to realize the large-scale production of *Cerasus humilis* and improve the nutritional quality of *Cerasus humilis*.

## 2 Materials and methods

### 2.1 Experiment materials

The experiment was conducted in the plastic greenhouse at the experimental base of Jilin Agricultural University in 2025. The experimental site is situated on a plain at 125°35’E, 43°82’N, falling within the northern temperate continental monsoon climate zone. Summers are hot and rainy, while winters are cold and dry. The average annual temperature is 4.5 °C, the average annual precipitation is approximately 600 mm, and the frost-free period lasts about 145 days.

**Figure 1:**
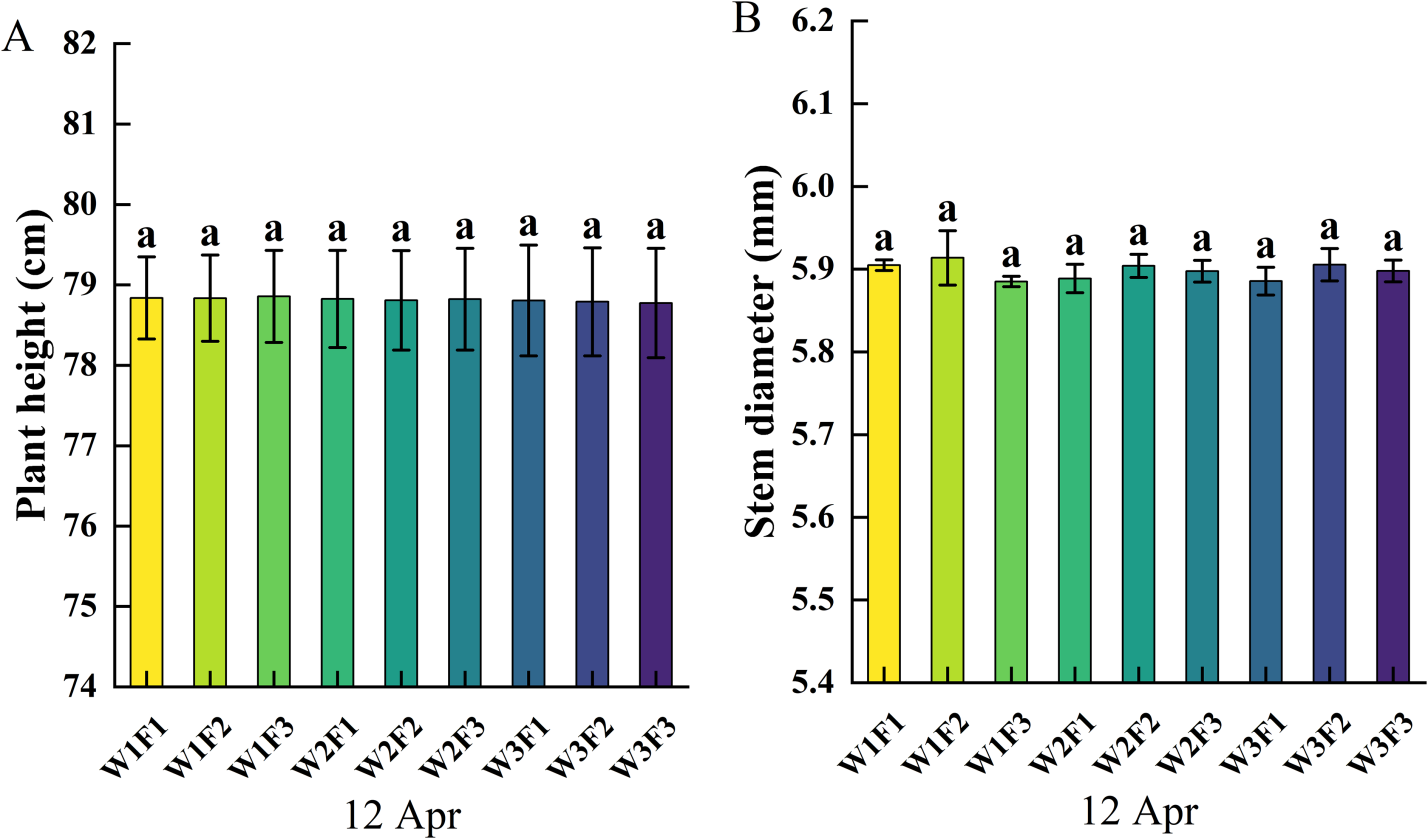
Plant height and stem diameter of different treatments before experiment: **(A)** plant height; **(B)** stem diameter, the same letter indicates no significant difference between different treatments (*p* > 0.05).

Three-year-old *Cerasus humilis* plants were used as test materials. The variety was ‘ Nongda No.6 ‘. The row spacing was 80 cm and the plant spacing was 60 cm. There were 9 treatments, 3 replicates, and 27 plots. Twenty plants with consistent growth were selected for planting in each plot. Before the experiment treatment (12 Apr), the plant height and stem diameter of different treatments are shown in **Figure 1**. The test soil was black loam. The basic physical and chemical properties of the 0–30 cm soil were as follows: soil bulk density 1.18 g/cm³, organic matter content 21.8 g/kg, alkali-hydrolyzable nitrogen 104.5 mg/kg, available phosphorus 148.2 mg/kg, available potassium 182 mg/kg, pH 6.52, field water holding capacity of root zone soil (FC) 32.7% (volumetric water content). The tested fertilizer was a compound fertilizer (N-P-K: 25-10-12, Jilin Longyuan Agricultural Service Co., Ltd.).

### 2.2 Experimental design

A two-factor completely randomized design was used in the experiment. The total drip irrigation amount was set at three levels: 60% FC (W1), 70% FC (W2), and 80% FC (W3). The total fertilization amount was set at three levels: F1 (66 kg/667m²), F2 (99 kg/667m²), and F3 (132 kg/667m²). There were 9 treatments in the experiment: W1F1, W1F2, W1F3, W2F1, W2F2, W2F3, W3F1, W3F2, and W3F3. To avoid mutual influence of water and fertilizer between different plots, a plastic film buried vertically at a depth of 60 cm was used for seepage prevention between the test plots. The field drip irrigation tubing was laid along the planting direction of *Cerasus humilis* plants. The drip tubing is an embedded cylindrical drip pipe with a diameter of 16 mm, a dripper flow rate of 1 L/h.

In this experiment, *Cerasus humilis* was planted on 20 Mar, and the fertilizer was first applied on 26 Apr. The fertilization stage was set at the embryonic stage, the fruit expansion stage, and the fruit color transition stage. During the embryonic stage, 40% of the total fertilizer was applied on 26 Apr, 30% was applied on 1 Jul during the fruit expansion stage, and 30% was applied on 30 Jul during the color transition stage. The first drip irrigation began on 13 Apr, and the drip irrigation was set at the embryonic stage, the young fruit stage, the fruit expansion stage, and the fruit color transition stage. The whole growth stage was dripped 10 times: the embryonic stage was dripped 3 times (13 Apr, 20 Apr, 26 Apr, respectively); The young fruit stage was dripped once on 10 Jun; the fruit expansion stage (25 Jun, 1 Jul, 8 Jul, 15 Jul, respectively) was dripped 4 times; and the fruit color transition stage (30 Jul, 10 Aug, respectively) was dripped 2 times. The specific amount of fertilizer is shown in **Table 1**, The amount of drip irrigation is shown in **Table 2**. The number of drip irrigation in each period is set according to the technical regulations of *Cerasus humilis* cultivation (Ma, 2023). The amount of drip irrigation is calculated according to the method of Li et al (Li et al., 2022), and the amount of fertilizer is set according to previous studies (Jiang, 2023).

**Table 1:** Fertilizer application rates at different growth stages(kg/667m²).

Treatments	Fertilization stage			Total fertilizer amount
	Embryonic stage (26 Apr)	Fruit expansion stage (1 Jul)	Fruit color transition stage (30 Jul)	
F1	26.4	19.8	19.8	66
F2	39.6	29.7	29.7	99
F3	52.8	39.6	39.6	132

**Table 2:** Times of irrigation and irrigation amount of drip irrigation at different growth stages(mm).

Treatments	Drip Irrigation amounts(mm)				Times of Irrigation in the embryonic stage	Times of Irrigation in the young fruit stage	Times of Irrigation in the fruit expansion stage	Times of Irrigation in the fruit color transition stage	Total drip irrigation amounts
	Embryonic stage(13 Apr、20 Apr、26 Apr)	Young fruit stage(10 Jun)	Fruit expansion stage(25 Jun、1 Jul、8 Jul、15 Jul)	Fruit color transition stage(30 Jul、10 Aug)					
W1	40.07	7.65	46.30	34.17	3	1	4	2	128.20
W2	59.80	18.36	65.84	36.72	3	1	4	2	180.73
W3	77.79	26.86	90.86	48.71	3	1	4	2	244.21

### 2.3 Determination items and methods

Starting from 1 Jun, the indicators were measured every 15 days, for a total of 6 times.

#### 2.3.1 Determination of growth indexes

Three replicate plots were set up in each treatment, and three plants were randomly selected in each plot for measurement. Finally, the average value of three replicates represented the test results of each treatment (Wang, 2024).

Plant Height: The plant height of *Cerasus humilis* was measured using a tape measure.

Stem Diameter: The stem diameter of *Cerasus humilis* was measured. For stems with a base diameter greater than 1 cm, a vernier caliper was used for measurement.

#### 2.3.2 Determination of photosynthetic indexes

Photosynthesis was measured using an LI-6400XTR plant photosynthesis analyzer. The net photosynthetic rate (Pn), transpiration rate (Tr), intercellular CO_2_ concentration (Ci), and stomatal conductance (Gs) were measured. The specific measurement time was 9:00-11:00 AM, selecting healthy leaves with good growth from the middle and upper parts of the plant for determination. Each treatment was repeated three times.

#### 2.3.3 Determination of fruit morphological traits

Three replicates were set for each treatment, and ten fruits were randomly selected from each replicate plot for determination. The average value of three replicates was used as the test result of this treatment (Yang et al., 2024).

Fruit fresh weight: Freshly harvested *Cerasus humilis* fruits were weighed using an analytical balance;

Fruit dry weight: dried in an oven and then weighed with an analytical balance;

Fruit horizontal and vertical diameters: measured using a vernier caliper.

#### 2.3.4 Determination of yield

Three replicate plots were set up for each treatment. At the fruit maturity stage, three plants were randomly selected in each plot to determine the yield and calculate the yield per plant. According to the plant spacing and row spacing, the number of plants planted per mu was calculated, and the yield per mu was obtained by multiplying the number of plants per mu by the yield per plant. The test results of each treatment were expressed as the average of three replicates (Wang, 2024).

#### 2.3.5 Determination of quality indexes

The content of soluble sugar was determined by the anthrone colorimetric method (Zhang and Chen, 2008), the content of titratable acid was determined by NaOH titration (Zhang and Chen, 2008), the content of soluble protein was determined using Coomassie Brilliant Blue G-250 (Zhang and Chen, 2008), the content of vitamin C was determined by spectrophotometry (Zhang and Chen, 2008), and the content of soluble solids was measured with a handheld sugar meter.

#### 2.3.6 Water and fertilizer utilization indexes

Water and fertilizer use efficiency is characterized by two indexes: irrigation water use efficiency (IWUE) and partial factor productivity (PFP). The calculation formula of each index is as follows (Equations 1, 2):

(1) $$IWUE=\frac{\text{Y}}{\text{I}}$$

(2) $$PFP=\frac{\text{Y}}{\text{F}}$$

In the equation: Y is yield (kg/677m^2^); I is the total irrigation water (mm) during the planting period; F is the total amount of fertilizer applied during the planting period (kg/677m^2^).

### 2.4 Data processing and analysis

The experimental data were processed and analyzed by Microsoft Excel 2016, SPSS 19.0 statistical software and origin2021. The Tukey method was used to test the significant difference between the treatments (*p* < 0.05). For variables measured multiple times on different dates, we performed analysis of variance by date. Two-way analysis of variance (ANOVA) was used to test the interaction of water and fertilizer coupling on Cerasus humilis (*p* < 0.05).

## 3 Results

### 3.1 Effects of water and fertilizer coupling on the growth of *Cerasus humilis*

#### 3.1.1 Effects of water and fertilizer coupling on the plant height of *Cerasus humilis*

**Figure 2:**
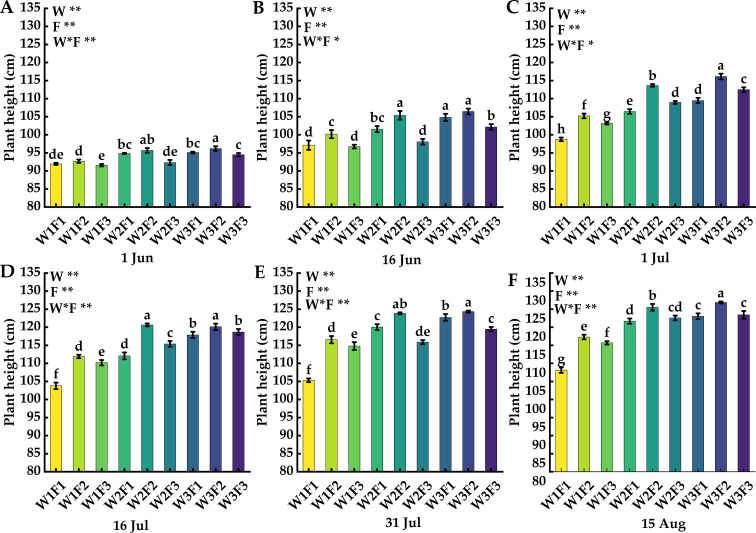
Effects of water and fertilizer coupling on the plant height of *Cerasus humilis* at different periods: **(A)** 1 Jun; **(B)** 16 Jun; **(C)** 1 Jul; **(D)** 16 Jul; **(E)** 31 Jul; **(F)** 15 Aug. Different letters indicate significant differences between treatments (*p* < 0.05). * indicates a highly significant difference at the *p* < 0.05 level, ** indicates a highly significant difference at the *p* < 0.01 level, and ns indicates no significant difference. W, F and W * F represent irrigation, fertilization factors and their interactions, respectively., the same below.

The effect of water and fertilizer coupling on the plant height of Cerasus humilis is shown in **Figure 2**. On 1 Jun, the plant height of W2F2 and W3F2 treatments was higher than that of other treatments, and the plant height of W1F3 treatment was the lowest; On 16 Jun, the plant height of W2F2, W3F1 and W3F2 treatments was significantly higher than that of other treatments, and there was no significant difference between the three treatments (*p* > 0.05); On 15 Aug, the plant height of W2F2 and W3F2 treatments was significantly higher than that of other treatments (*p* < 0.05), and the difference between the two treatments also reached a significant level, the plant height of W1F1 treatment was the lowest.

#### 3.1.2 Effects of water and fertilizer coupling on the stem diameter of *Cerasus humilis*

**Figure 3:**
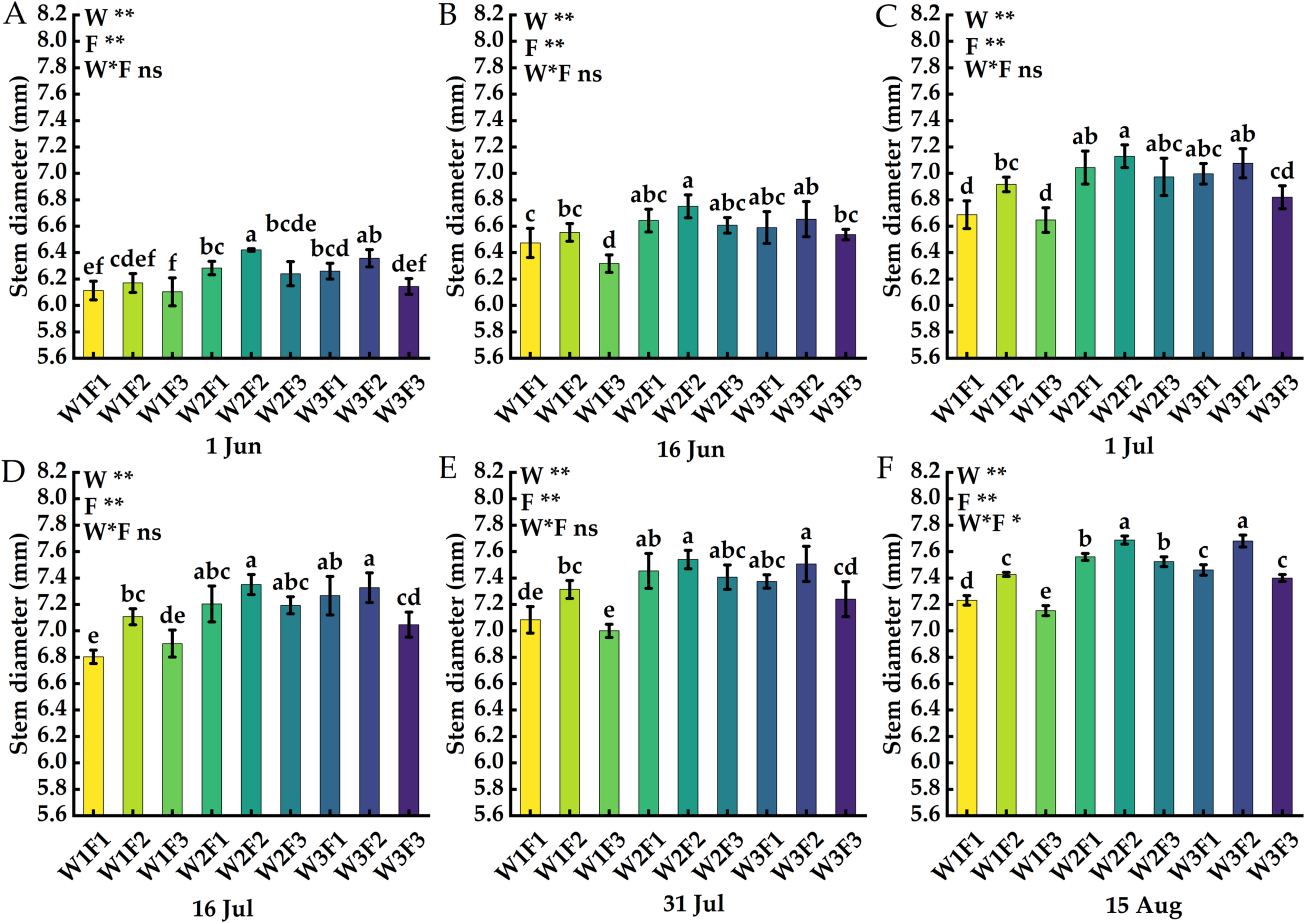
Effects of water and fertilizer coupling on stem diameter of *Cerasus humilis* at different periods: **(A)** 1 Jun; **(B)** 16 Jun; **(C)** 1 Jul; **(D)** 16 Jul; **(E)** 31 Jul; **(F)** 15 Aug. Different letters indicate significant differences between treatments (*p <* 0.05). * indicates a highly significant difference at the *p <* 0.05 level, ** indicates a highly significant difference at the *p < 0.01* level, and ns indicates no significant difference. W, F and W * F represent irrigation, fertilization factors and their interactions, respectively.

The effect of water and fertilizer coupling on the stem diameter of *Cerasus humilis* is shown in **Figure 3**. On 1 Jun, W2F2 and W1F3 reached the highest and lowest values respectively; On 16 Jun, the stem diameter of plants treated with W2F1, W2F2, W2F3, W3F1 and W3F2 was significantly higher than that of other treatments, and there was no significant difference between the five treatments (*p* > 0.05); On 15 Aug, the maximum of stem diameter (W2F2) increased by 7.55% compared with the lowest value (W1F3), and there was a significant difference, the stem diameter of *Cerasus humilis* in W2F2 treatment increased by 19.78%, 13.93%, 7.85%, 4.63% and 1.99%, respectively, compared with the first five periods, the W1F3 treatment increased by 17.21%, 13.13%, 7.52%, 3.62% and 2.14%, respectively, compared with the first five periods. The results showed that appropriate irrigation combined with compound fertilizer application could promote the growth of *Cerasus humilis*.

### 3.2 Effects of water and fertilizer coupling on photosynthesis of leaves of *Cerasus humilis* at different stages

In this experiment, Jul is the stage of *Cerasus humilis* fruit formation. This stage is a key turning point in the distribution of photosynthetic products. The strength of leaf photosynthetic indicators directly determines the transport efficiency of photosynthetic products to the fruit, which in turn affects the subsequent fruit enlargement and quality construction. It is the core period to reflect the photosynthetic regulation effect of water and fertilizer coupling on the reproductive growth of *Cerasus humilis*. From the perspective of data characteristics, the above four photosynthetic indexes in this study all reached the extreme value in the determination period on 1 Jul.

**Figure 4:**
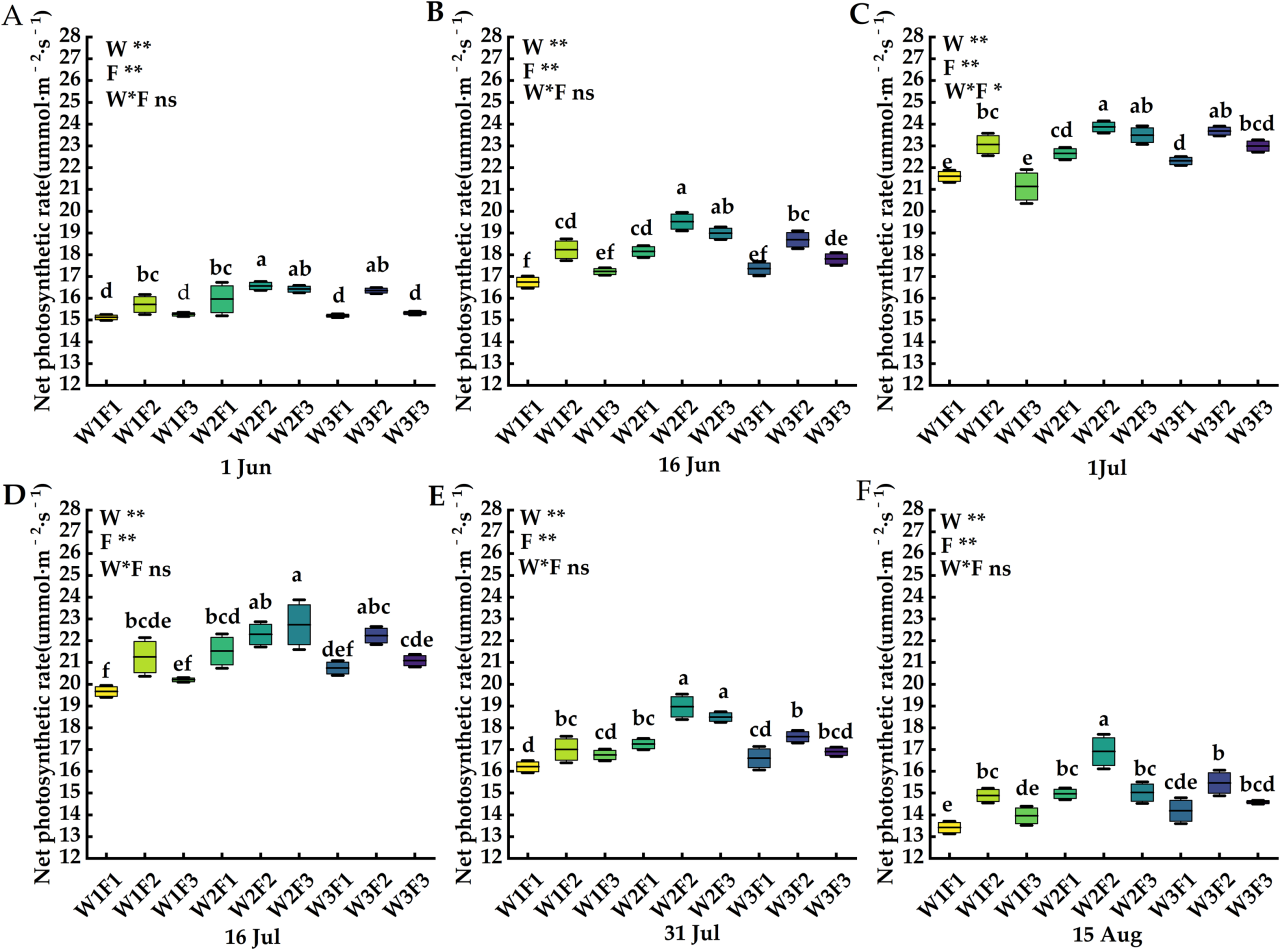
Effects of water and fertilizer coupling on the net photosynthetic rate of leaves of *Cerasus humilis* at different stages: **(A)** 1 Jun; **(B)** 16 Jun; **(C)** 1 Jul; **(D)** 16 Jul; **(E)** 31 Jul; **(F)** 15 Aug. Different letters indicate significant differences between treatments (*p <* 0.05). * indicates a highly significant difference at the *p <* 0.05 level, ** indicates a highly significant difference at the *p <* 0.01 level, and ns indicates no significant difference. W, F and W * F represent irrigation, fertilization factors and their interactions, respectively. The 9 boxes in each figure represent different treatments, the horizontal line in the middle of the box represents the average value of each treatments, and the upper and lower error bars represent the maximum and minimum values of the values in the group.

**Figure 5:**
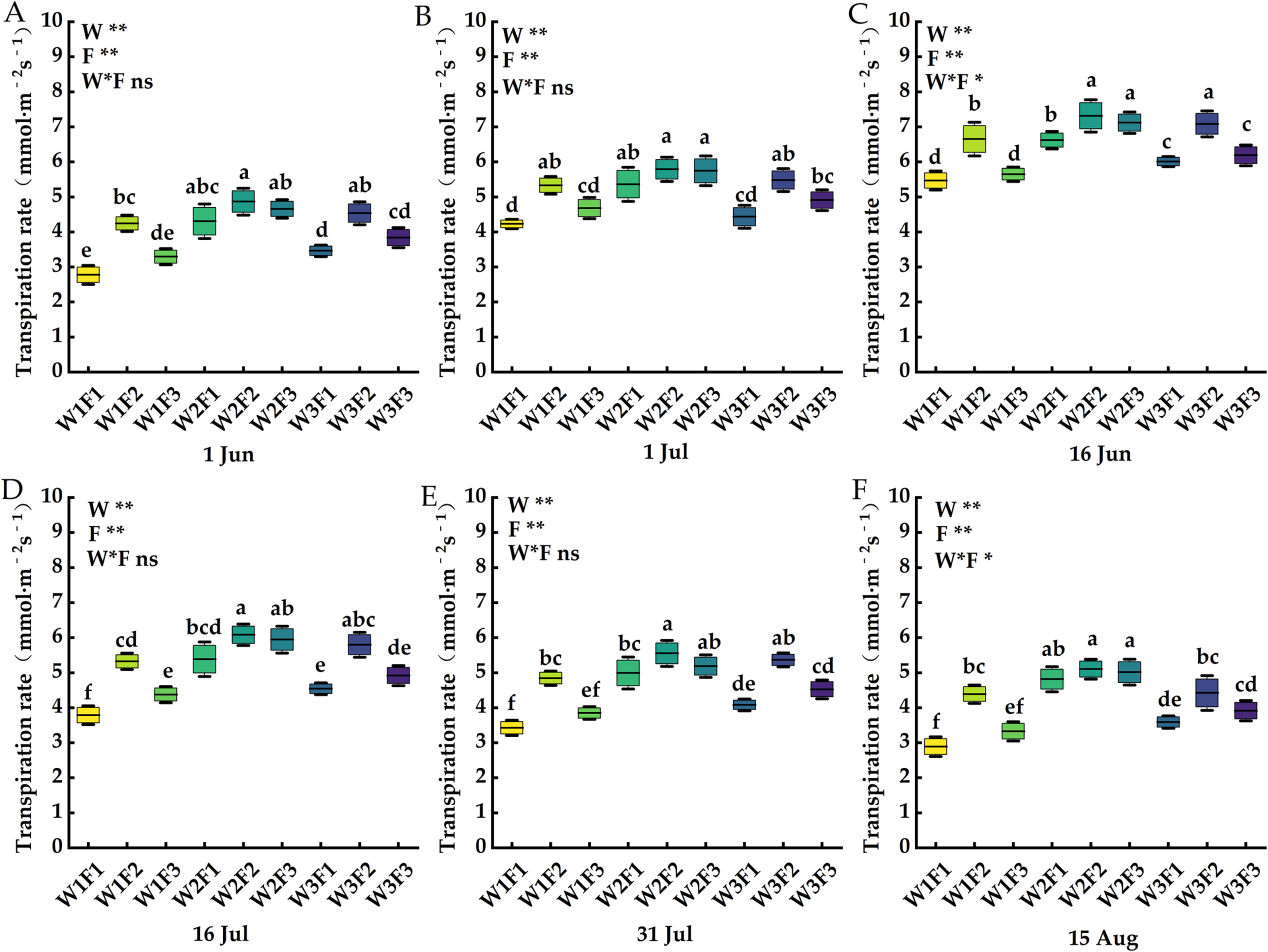
Effects of water and fertilizer coupling on the Transpiration rate of leaves of *Cerasus humilis* at different stages: **(A)** 1 Jun; **(B)** 16 Jun; **(C)** 1 Jul; **(D)** 16 Jul; **(E)** 31 Jul; **(F)** 15 Aug. Different letters indicate significant differences between treatments (*p <* 0.05). * indicates a highly significant difference at the *p < *0.05 level, ** indicates a highly significant difference at the *p <* 0.01 level, and ns indicates no significant difference. W, F and W * F represent irrigation, fertilization factors and their interactions, respectively. The 9 boxes in each figure represent different treatments, the horizontal line in the middle of the box represents the average value of each treatments, and the upper and lower error bars represent the maximum and minimum values of the values in the group.

**Figure 6:**
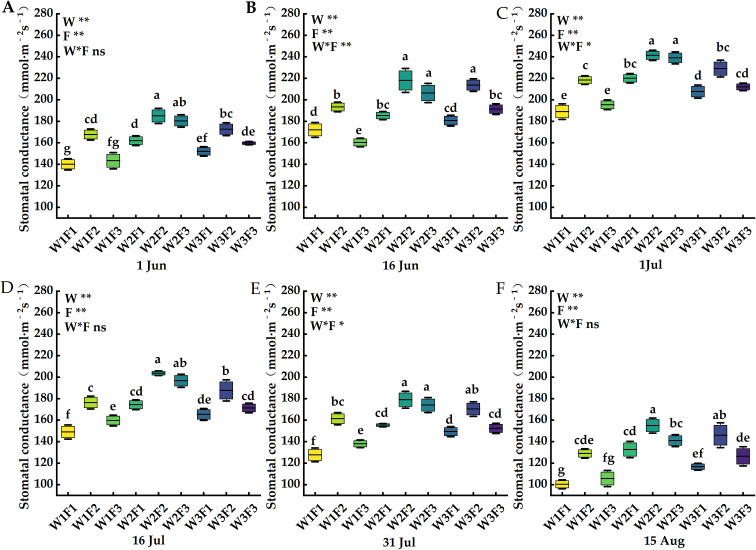
Effects of water and fertilizer coupling on the Stomatal conductance of leaves of *Cerasus humilis* at different stages: **(A)** 1 Jun; **(B)** 16 Jun; **(C)** 1 Jul; **(D)** 16 Jul; **(E)** 31 Jul; **(F)** 15 Aug. Different letters indicate significant differences between treatments (*p <* 0.05). * indicates a highly significant difference at the *p <* 0.05 level, ** indicates a highly significant difference at the *p <* 0.01 level, and ns indicates no significant difference. W, F and W * F represent irrigation, fertilization factors and their interactions, respectively. The 9 boxes in each figure represent different treatments, the horizontal line in the middle of the box represents the average value of each treatments, and the upper and lower error bars represent the maximum and minimum values of the values in the group.

**Figure 7:**
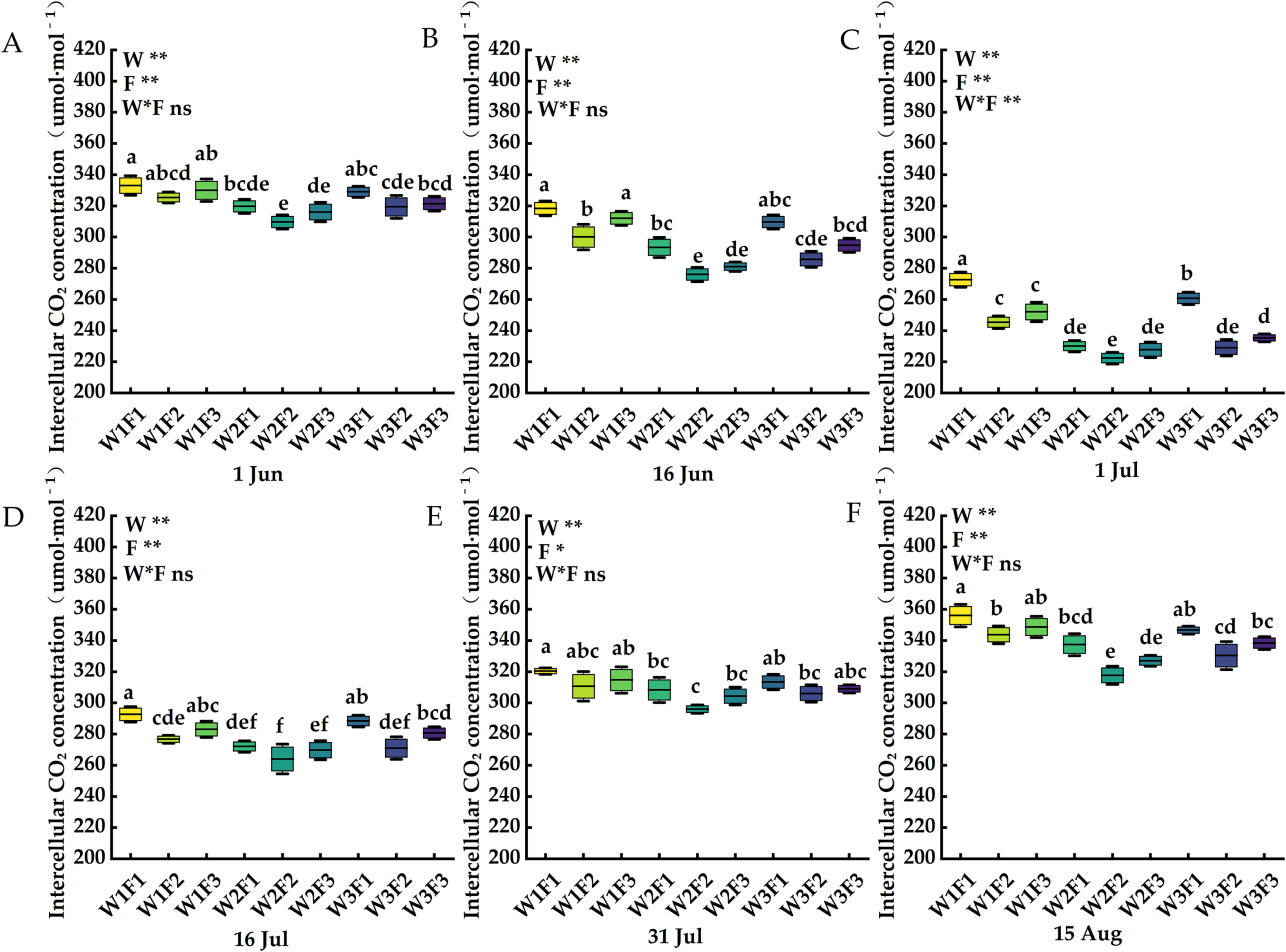
Effects of water and fertilizer coupling on the Intercellular carbon dioxide concentration of leaves of *Cerasus humilis* at different stages: **(A)** 1 Jun; **(B)** 16 Jun; **(C)** 1 Jul; **(D)** 16 Jul; **(E)** 31 Jul; **(F)** 15 Aug. Different letters indicate significant differences between treatments (*p <* 0.05). * indicates a highly significant difference at the *p <* 0.05 level, ** indicates a highly significant difference at the *p <* 0.01 level, and ns indicates no significant difference. W, F and W * F represent irrigation, fertilization factors and their interactions, respectively. The 9 boxes in each figure represent different treatments, the horizontal line in the middle of the box represents the average value of each treatments, and the upper and lower error bars represent the maximum and minimum values of the values in the group.

The effects of water and fertilizer coupling on net photosynthetic rate, transpiration rate, stomatal conductance and intercellular carbon dioxide concentration of *Cerasus humilis* leaves are shown in **Figures 4**–**7**. On 1 Jul, the net photosynthetic rate, transpiration rate and stomatal conductance of W2F2 treatment were the largest, and the net photosynthetic rate, transpiration rate and stomatal conductance of each period showed a trend of increasing first and then decreasing, and the intercellular carbon dioxide concentration showed a trend of decreasing first and then increasing. In terms of transpiration rate, compared with 1 Jun and 16 Jun, the transpiration rate of *Cerasus humilis* leaves treated with W2F2 increased by 50.41% and 26.25% respectively, the W1F1 treatment increased by 97.47% and 29.31%, respectively; the change trend of stomatal conductance and net photosynthetic rate was similar to that of transpiration rate; the intercellular carbon dioxide concentration of *Cerasus humilis* leaves treated with W1F1 reached the maximum value of 272.67μmol·mol^-1^, which was 22.13% and 16.75% lower than that on 1 Jun and 16 Jun, respectively, W2F2 treatment decreased by 39.28% and 24.14%, respectively. The results showed that the reasonable combination of water and fertilizer treatment was beneficial to promote the photosynthesis of *Cerasus humilis*.

### 3.3 Effects of water and fertilizer coupling on fruit morphology and yield of *Cerasus humilis*

As shown in **Table 3**, water and fertilizer coupling had a significant effect on the fresh weight and yield of *Cerasus humilis* fruit (*p* < 0.05). The dry and fresh weight, horizontal and vertical diameter, yield, and other indicators of *Cerasus humilis* reached their maximum under the W2F2 treatment, and the yield of W2F2 was 61.78% higher than that of the lowest-yielding W1F1. There was no significant difference in fruit fresh weight between the W2F2 and W2F3 treatments, both of which were significantly higher than those of the other treatment groups (*p* < 0.05).

**Table 3:** Significance analysis of water and fertilizer coupling on fruit morphological development, water and fertilizer utilization rate and yield of *Cerasus humilis* (F value).

F Value	Fruit horizontal diameter(mm)	Fruit vertical diameter(mm)	Single fruit fresh weight(g)	Single fruit dry weight(g)	IWUE	PFP	Yield(kg/667m²)
W	27.50**	29.51**	426.64**	27.15**	987.91**	331.19**	399.87**
F	8.77**	6.23**	235.45**	18.02**	217.46**	1121.36**	257.97**
W*F	1.39ns	2.10ns	16.40**	1.02ns	12.57**	6.67**	10.04**

It can be seen from **Table 4** that different water and fertilizer treatments had a significant impact on fruit morphological indexes. For the vertical diameter of the fruit, the W2F2 treatment was the largest, reaching 17.62 mm, while the W1F1 treatment was the smallest, at 14.84 mm. There was a significant difference between the two (*p* < 0.05). Comparing the horizontal diameter of fruits under different treatments, the W2F2 treatment had the largest horizontal diameter of 18.80 mm, followed by the W2F3 treatment.

**Table 4:** Effects of different water and fertilizer coupling on fruit morphology and yield of *Cerasus humilis*.

Treatments	Fruit horizontal diameter(mm)	Fruit vertical diameter(mm)	Single fruit fresh weight(g)	Single fruit dry weight(g)	Yield(kg/667m²)
W1F1	15.88 ± 0.2f	14.84 ± 0.52d	2.66 ± 0.1f	0.38 ± 0.02e	685.81 ± 25.68f
W1F2	17.02 ± 0.45cde	16.00 ± 0.48bc	3.22 ± 0.08d	0.45 ± 0.04cd	872.31 ± 20.18d
W1F3	16.06 ± 0.78ef	14.92 ± 0.47d	2.77 ± 0.05ef	0.39 ± 0.04de	726.67 ± 11.93e
W2F1	17.53 ± 0.30bcd	16.61 ± 0.37abc	3.48 ± 0.03c	0.46 ± 0.02cd	902.91 ± 19.72d
W2F2	18.80 ± 0.49a	17.62 ± 0.72a	4.16 ± 0.07a	0.58 ± 0.06a	1109.49 ± 29.04a
W2F3	18.51 ± 0.28ab	17.50 ± 0.54a	4.06 ± 0.1a	0.55 ± 0.03a	1063.26 ± 16.01b
W3F1	16.54 ± 0.42def	15.79 ± 0.70cd	2.87 ± 0.07e	0.43 ± 0.02cde	749.83 ± 9.18e
W3F2	17.33 ± 0.80cd	16.21 ± 0.64bc	3.88 ± 0.06b	0.53 ± 0.04ab	991.06 ± 15.40c
W3F3	17.79 ± 0.88abc	16.92 ± 0.40ab	3.53 ± 0.1c	0.48 ± 0.03bc	905.84 ± 22.56d

### 3.4 Effects of water and fertilizer coupling on fruit quality

**Figure 8:**
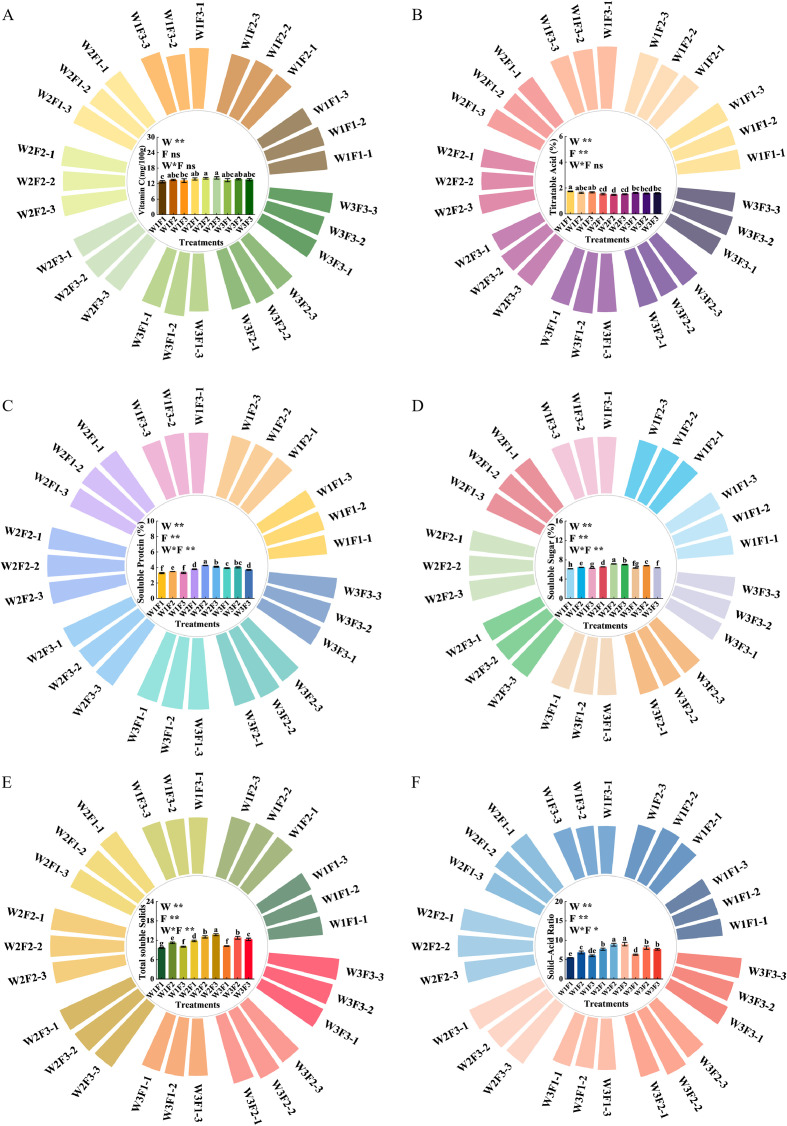
Effects of different combinations of water and fertilizer on the content of soluble sugar, soluble protein, vitamin C and titratable acid in *Cerasus humilis*: **(A)** vitamin C; **(B)** titratable acid; **(C)** soluble protein; **(D)** soluble sugar; **(E)** total soluble solids; **(F)** solid–acid ratio. The three columns of the same color in each image represent the three repeated values of each treatment.

The effects of water and fertilizer interaction on the fruit quality of *Cerasus humilis* is shown in **Figure 8**. Different water and fertilizer treatments significantly increased the content of soluble sugars and soluble proteins in *Cerasus humilis*. The soluble sugar content in the W2F2 treatment was significantly different from that of the other treatments, reaching a maximum of 7.14%, with the W2F2 treatment group being 15.91% higher than W1F1. There was no significant difference between W3F1 and W3F3. The change in soluble protein was consistent with that of soluble sugar, reaching its maximum in the W2F2 treatment, which was significantly higher than in the other treatment groups. The W2F2 treatment group showed an increase of 29.57% compared with W1F1. There was no significant difference in titratable acid content among W2F1, W2F2, and W2F3, and the titratable acid content of W1F1 in the treatment group was the highest (1.75%), while W2F2 was the lowest (1.47%), The W1F1 treatment group increased by 19.05% compared to W2F2. The VC content reached its highest value (14.20 mg/100g) in the W2F3 treatment, which was significantly higher than in W1F1 and W1F3 treatments, and showed no significant difference compared with other treatments, the W2F3 treatment group increased by 11.46% compared with W1F1. The soluble solids content reached its maximum (13.70%) in the W2F3 treatment, which was significantly higher than in other treatments. The solid-acid ratio reached a maximum value of 9.02 in the W2F3 treatment, which was not significantly different from the W2F2 treatment. The interaction between water and fertilizer had a highly significant effect on soluble protein, soluble sugar, soluble solids, and the solid-acid ratio of *Cerasus humilis*, but had no significant effect on titratable acid and vitamin C (*p* < 0.05).

### 3.5 Effects of water and fertilizer coupling on water and fertilizer use efficiency of *Cerasus humilis*

**Figure 9:**
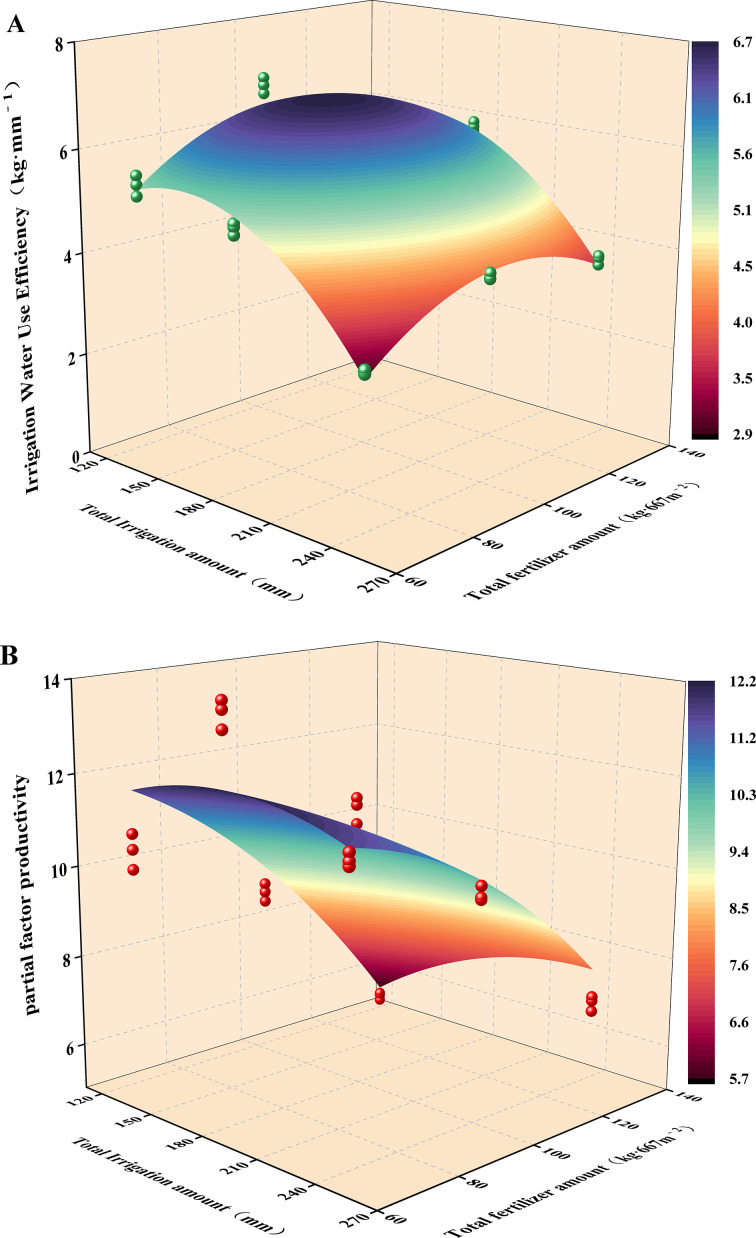
Regression model of water and fertilizer use efficiency of *Cerasus humilis*: **(A)** irrigation water use efficiency; **(B)** partial factor productivity.

The effects of water and fertilizer coupling on IWUE and PFP of *Cerasus humilis* were shown in **Figure 9**. The results showed that water and fertilizer coupling significantly affected IWUE and PFP of *Cerasus humilis* (*p* < 0.05), as shown in **Table 3**. IWUE was negatively correlated with the increase of irrigation amount at F1 and F2 levels and decreased with the increase of irrigation amount at F3 level. W1F2 and W2F2 treatments were the highest, significantly better than other treatments, which were 27.10% and 14.77% higher than W1F1 treatment, respectively. PFP increased first and then decreased with the increase of irrigation amount and was negatively correlated with fertilization amount. PFP reached the maximum value under W2F1 treatment, followed by W2F2 treatment, which increased by 31.67% and 7.89% compared with W1F1 treatment. In summary, increasing the amount of irrigation and fertilization can improve the utilization efficiency of water and fertilizer. And taking irrigation amount and compound fertilizer application amount as independent variables, irrigation water use efficiency and partial factor productivity of *Cerasus humilis* as dependent variables, a regression model fitting surface was constructed. The F values of the model were 155.25 and 32.87, (*p* < 0.05), indicating that the model was significant. The regression model shows that a moderate increase in water and fertilizer may improve overall productivity, while excessive water or fertilizer input will reduce marginal efficiency.

### 3.6 Principal component analysis of yield、quality, water and fertilizer utilization rate of *Cerasus humilis*

#### 3.6.1 Principal component analysis

In order to consider the possible impact of different dimensions, the original data has been standardized before principal component analysis (Yang et al., 2022). According to the principle of eigenvalue greater than 1, two principal components were extracted from the indexes of yield, quality and water and fertilizer utilization rate. The contribution rate of the first principal component was 71.81%, and the contribution rate of the second principal component was 10.09%. These indicators include fruit transverse stem, longitudinal stem, single fruit dry weight, single fruit fresh weight, total yield, soluble solids, soluble protein, soluble sugar, titratable acid, vitamin C, solid acid ratio, irrigation water use efficiency, fertilizer partial factor productivity. A total of 13 indicators were analyzed by principal component analysis. The contribution rate of principal component analysis is shown in **Table 5**. It can be seen that the extracted principal components explain 81.90% of the total variance, and the value is greater than 80%, indicating that the selection of two principal components can explain the problem.

**Table 5:** Load matrix, eigenvalues, and (cumulative) contribution rate of each principal component factor.

Indicators		PCA1	PCA2
Fator loading	Yield	0.978	0.107
	Single Fruit Fresh Weight	0.971	0.052
	Single Fruit Dry Weight	0.916	0.036
	Fruit Horizontal Diameter	0.917	0.063
	Fruit Vertical Diameter	0.889	−0.025
	Soluble Sugar	0.944	0.090
	Total Soluble Solids	0.958	0.086
	Titratable Acid	−0.881	0.109
	Vitamin C	0.777	0.024
	Soluble protein	0.870	−0.376
	Solid–Acid Ratio	0.977	0.037
	IWUE	0.152	0.795
	PFP	0.204	−0.7
Eigenvalues		9.34	1.31
Contribution Rate (%)		71.81%	10.09%
Cumulative Contribution (%)		71.81%	81.90%

#### 3.6.2 Comprehensive evaluation of *Cerasus humilis* based on principal component analysis

In order to more intuitively describe the relationship between the main components and their related yield, quality, water and fertilizer utilization rate, a linear relationship equation was constructed to achieve comprehensive evaluation by using indicators with high correlation. In this equation, Y represents the score of this major component. The higher the Y value, the better the growth of *Cerasus humilis*.

(3) $$Y_{1}=0.309X_{1}-0.288X_{2}+0.313X_{3}+0.285X_{4}+0.320X_{5}+0.254X_{6}+0.318X_{7}+0.3X_{8}+0.3X_{9}+0.291X_{10}+0.32X_{11}+0.05X_{12}+0.067X_{13}$$

(4) $$Y_{1}=0.029X_{1}+0.036X_{2}+0.028X_{3}-0.123X_{4}+0.012X_{5}+0.008X_{6}+0.017X_{7}+0.021X_{8}+0.012X_{9}-0.008X_{10}+0.035X_{11}+0.260X_{12}-0.229X_{13}$$

(5) $$Y=0.877Y_{1}+0.123Y_{2}$$

In the formula, X_1_ ~ X_13_ represent soluble sugar, titratable acid, soluble solids, soluble protein, solid-acid ratio, vitamin C, single fruit fresh weight, fruit horizontal diameter, single fruit dry weight, fruit vertical diameter, yield, irrigation water use efficiency, and partial factor productivity. Based on various calculation results, the comprehensive score and ranking are determined, Y1 and Y2 represent the scores of the two principal components, respectively, and Y is the final selection of principal component score and comprehensive ranking basis (Equations 3–5).

The results in **Table 5** showed that the main influencing factors of PCA1 were yield, fruit size and fruit quality, which were significantly correlated with PCA1, indicating that the greater the principal component load, the greater the index. The main influencing factors of PCA2 are IWUE and PFP, which are significantly correlated with PCA2, indicating that the greater the principal component load value, the greater the two indicators.

By calculating the comprehensive score and sorting it, the comprehensive evaluation results of different water and fertilizer coupling schemes were obtained (**Table 6**). The comprehensive score of W2F2 treatment was the first, indicating that the treatment was relatively good. The higher the comprehensive score of PCA, the more balanced the yield, fruit quality, photosynthetic performance, irrigation water use efficiency and partial factor productivity of Cerasus humilis. The comprehensive score is positive, indicating that the growth, yield and water and fertilizer utilization of *Cerasus humilis* are higher than the average.

**Table 6:** Comprehensive score of principal components.

Treatments	Y1	Y2	Y	Order
W1F1	-4.66772499	0.01840795	-4.091330638	9
W1F2	-1.09886651	0.44755367	-0.908656828	6
W1F3	-3.6917879	0.49147198	-3.177246935	8
W2F1	0.78971971	-0.4470922	0.637591845	4
W2F2	4.64987559	0.08459066	4.088345544	1
W2F3	3.97399237	0.29493495	3.521468307	2
W3F1	-2.00628471	-0.5874874	-1.831772641	7
W3F2	1.74147143	-0.23109313	1.498845989	3
W3F3	0.30961133	-0.07128886	0.262760607	5

## 4 Discussion

*Cerasus humilis* is a perennial fruit tree, and the growth index is the most intuitive representation of its growth and development. In actual agricultural production, due to the complex interactions between *Cerasus humilis* and water and nutrient supply, the growth and development of *Cerasus humilis* require suitable water and fertilizer conditions. An appropriate amount of irrigation and fertilization can effectively promote the healthy growth of the crop (Zhou et al., 2023). In this experiment, different combinations of water and fertilizer had a positive effect on the growth of *Cerasus humilis* plants. The combined regulation of water and fertilizer could significantly promote the growth of *Cerasus humilis*. Similar patterns have also been observed in watermelon-related research (Hong et al., 2022). This study indicates that plant growth is inhibited under low water conditions, which may be related to the loss of cell turgor pressure, subsequently affecting cell division, elongation, and differentiation (Blackman, 2018). Wang et al (Wang, 2016) showed that excessive fertilization and irrigation could significantly inhibit the growth of grape stem diameter, restricting plant development. The results of this experiment were consistent with those of Wang et al. This may be due to excessive soil moisture, which reduces the oxygen content in the plant rhizosphere, makes root respiration difficult, and decreases root activity (Pedersen et al., 2013; van Veen et al., 2013).

Plant photosynthetic physiology is an important manifestation of the physiological and biochemical processes of crops. Therefore, plant growth and development are also influenced by photosynthetic characteristics, which are regulated by irrigation and fertilization. Consequently, photosynthetic characteristics are key indicators for evaluating the overall benefits of irrigation and fertilization management. Photosynthetic parameters such as photosynthetic rate (Pn), transpiration rate (Tr), intercellular carbon dioxide concentration (Ci), and stomatal conductance (Gs) are important factors affecting crop growth and yield, and form the basis of crop metabolism (Zhou et al., 2023; Zhang et al., 2017). Hong et al. (2022) demonstrated that an appropriate amount of irrigation can protect the photosynthetic organs of crops and enhance photosynthetic capacity, this may be due to the fact that a good soil moisture environment can promote the opening of leaf stomata, increase the absorption of CO_2_ by leaves, enhance the transport of photosynthetic products, and reduce the inhibition of photosynthesis caused by the accumulation of photosynthetic products in leaves (Abdalla et al., 2021; Ma et al., 2019). Studies have also shown that soil water deficit induces plant roots to produce abscisic acid, which sends inhibitory signals and restrains plant growth. These signals are transmitted to the canopy through the xylem. Under the influence of water transfer, the stomata in the canopy close, leading to a decrease in the photosynthetic rate (Dos Santos et al., 2015; Wei et al., 2022). Li et al. (2021) found that increased fertilization significantly enhanced the photosynthetic capacity of seedling mango trees, but excessive fertilization had an inhibitory effect, which aligns with the results of this experiment, this may be because excessive fertilizer will increase the osmotic potential of soil solution, thereby reducing the water potential gradient between soil solution and root system, reducing the absorption of water and nutrients by root system, resulting in a decrease in root water absorption capacity, causing water and nutrient stress, resulting in a decrease in photosynthetic efficiency (Hong et al., 2022; Zhou et al., 2020).

Fruit yield are key indicators of economic value. This experiment showed that different combinations of water and fertilizer had significant effects on the yield of *Cerasus humilis*. Wang et al. showed that within a certain range of water and fertilizer input, yield increased with the increase in irrigation and fertilizer application, but when the amount of irrigation and fertilizer was below or above a certain threshold, yield decreased, this may be due to low levels of water and fertilizer, which result in shorter plants and smaller leaves, leading to lower LAI, photosynthetic efficiency is weak under low LAI, which is not conducive to high yield, increasing irrigation can improve LAI, dry matter accumulation, and yield (Wang et al., 2018; Wei et al., 2022). Studies have also shown that excessive use of nitrogen and phosphate fertilizers can reduce fruit yield (Zhang et al., 2024a). Li et al. (2010) found that yield increased with increased fertilizer application, but excessive fertilization did not further increase yield, and we also reached the same conclusion. Shareef et al. found (Shareef et al., 2018) that with an increase in irrigation, yield improved, which is similar to the findings of this experiment.

Soluble sugar, soluble protein, soluble solids, titratable acid, solid-acid ratio, and vitamin C are key indicators of *Cerasus humilis* quality. Soluble sugar is a direct product of plant photosynthesis, while soluble protein is an important osmotic regulator and nutrient for plants. Both play vital roles in plant growth and development, adaptation to environmental changes, nutrient accumulation, and transport, and are important indicators reflecting the physiological status of plants (Sun and Liu, 2018; Zhang et al., 2009). Non-volatile flavor-related compounds in fruits mainly include sugars and acids. The perception of sweetness and flavor is determined by soluble sugars, organic acids, and their ratios (Usenik et al., 2010). Yu et al. (2023) showed that excessive use of fertilizer can hinder the absorption of other minerals such as potassium, calcium, and magnesium, which are important for fruit quality. Zhang et al. (2024b) found that the content of soluble sugar in fruit increased with the increase of nitrogen fertilizer. An excessive increase in phosphate fertilizer reduced the content of soluble sugar in fruit, which is similar to the conclusion of Zhou et al. (2015). Yang et al. (2020) showed that deficit irrigation could increase the content of soluble sugar, which is similar to the results of this experiment. This may be because moderate reduction in irrigation can increase the activities of acid invertase, neutral invertase, and sucrose synthase, thereby promoting the synthesis of soluble sugar (Yang et al., 2015). Vitamin C is a very important vitamin and an essential substance required by the human body. Since the human body cannot synthesize vitamin C, it is considered an essential dietary micronutrient that must be obtained through diet (Luo et al., 2022). Studies have shown that moderate watering can increase the accumulation of vitamin C (Vc) in citrus (Chen et al., 2022), Similar to the results of this experiment, this phenomenon may be due to increased water accumulation leading to the dilution of fruit components (Du et al., 2017), more sugar accumulation and less water are conducive to the synthesis of vitamin C (Veit-Köhler et al., 1999). Zhou et al. found that under moderate water deficit irrigation, the content of vitamin C in apples increased with the increase of nitrogen application rate, which was similar to the results of this experiment (Zhou et al., 2015). The results of this study showed that water and fertilizer coupling had a great influence on titratable acid. Parvizi and Sepaskhah (2015) showed that water stress increased the content of titratable acid compared with sufficient irrigation, which was consistent with the results of this experiment.

In this experiment, IWUE decreased with the increase of irrigation amount, and decreased with the increase of fertilization amount. It shows that high water content is not conducive to the absorption and utilization of water in *Cerasus humilis*. Under W1F2 treatment, IWUE reached the maximum, followed by W2F2 treatment, indicating that the appropriate water and fertilizer coupling scheme can improve the water absorption and utilization of Cerasus humilis plants. PFP increased first and then decreased with the increase of irrigation amount, and was negatively correlated with fertilization amount. Higher PFP does not necessarily lead to the highest yield (Zhang et al., 2018). This experiment confirmed this finding, because the results showed that compared with W2F2 treatment, W2F1 treatment had the highest PFP and the yield deficit was reduced. Nutrient deficiency causes *Cerasus humilis* plants to prioritize their own growth by absorbing more nutrients, resulting in reduced yield (Yue et al., 2023; He et al., 2023), it indicated that moderate increase of water and fertilizer could improve the overall productivity, while excessive water or fertilizer input would reduce the marginal efficiency. Overall, W2F2 treatment in this experiment provided the best overall balance between yield, fruit quality, photosynthetic performance, irrigation water use efficiency, partial factor productivity and comprehensive ranking based on principal component analysis, while higher water and fertilizer treatment showed relatively excessive vegetative growth and decreased yield and water and fertilizer use efficiency, indicating that excessive water and fertilizer input would disturb the balance between vegetative growth, fruit development and assimilation distribution. Therefore, in this experiment, the medium level of water and fertilizer was the most suitable for the water and fertilizer needs of *Cerasus humilis*.

In summary, this experiment studied the growth, photosynthesis, yield, quality and water and fertilizer utilization efficiency of *Cerasus humilis* under different water and fertilizer coupling management schemes. Principal component analysis was used to comprehensively evaluate the various yield and quality indicators, indicating that the comprehensive score of the W2F2 treatment was the highest. This treatment can serve as a reference for improving the quality and yield of *Cerasus humilis*. However, this study only carried out regulation research on key growth stages, and the revelation of nutrient demand rules at different stages was not deep enough, and it was carried out under a cultivation environment. In the future, we can combine data modeling and other methods, the accurate ratio of water and fertilizer in each growth period can be further quantified, in the future, it will be verified across years, locations and soil conditions, and then a more accurate local water and fertilizer scheme for *Cerasus humilis* can be proposed.

## 5 Conclusions

Through field tests on the coupling of water and fertilizer, the synergistic effect of water and fertilizer was comprehensively considered. By analyzing the effects of this coupling on the growth and development, fruit morphology, dry and fresh weight, photosynthesis, yield, quality and water and fertilizer utilization efficiency of *Cerasus humilis*, the response mechanism of water and fertilizer regulation was discussed. The results showed that W2F2 treatment showed advantages in yield, photosynthetic performance, soluble protein and soluble sugar, and W2F3 treatment showed advantages in vitamin C content and solid acid ratio quality index. Comprehensive analysis showed that W2F2 treatment provided the best overall balance between yield, fruit quality, photosynthetic performance, irrigation water use efficiency, partial factor productivity and comprehensive ranking based on principal component analysis. Therefore, W2F2 treatment is recommended as the best water and fertilizer coupling scheme for the comprehensive performance of *Cerasus humilis* production. The research results can provide reference for guiding the high-quality and efficient production of *Cerasus humilis* in facilities. In the future, we will verify and propose irrigation and fertilization strategies across multiple seasons and production environments for nutrient uptake or tissue nutrient analysis to better characterize real nutrient use efficiency.

## Data Availability

The original contributions presented in the study are included in the article. Further inquiries can be directed to the corresponding authors.
